# Development of a Disease-Specific Health-Related Quality of Life Questionnaire (DTF-QoL) for Patients with Desmoid-Type Fibromatosis

**DOI:** 10.3390/cancers14030709

**Published:** 2022-01-29

**Authors:** Anne-Rose W. Schut, Emma Lidington, Milea J. M. Timbergen, Eugenie Younger, Winette T. A. van der Graaf, Winan J. van Houdt, Johannes J. Bonenkamp, Robin L. Jones, Dirk. J. Grünhagen, Stefan Sleijfer, Cornelis Verhoef, Spyridon Gennatas, Olga Husson

**Affiliations:** 1 Department of Medical Oncology, Erasmus MC Cancer Institute, 3015 GD Rotterdam, The Netherlands; a.schut@erasmusmc.nl (A.-R.W.S.); m.timbergen@erasmusmc.nl (M.J.M.T.); w.vd.graaf@nki.nl (W.T.A.v.d.G.); s.sleijfer@erasmusmc.nl (S.S.); 2 Department of Surgical Oncology, Erasmus MC Cancer Institute, 3015 GD Rotterdam, The Netherlands; d.grunhagen@erasmusmc.nl (D.J.G.); c.verhoef@erasmusmc.nl (C.V.); 3 Sarcoma Unit, Royal Marsden NHS Foundation Trust, London SW3 6JJ, UK; emma.lidington@rmh.nhs.uk (E.L.); eugenie.younger@rmh.nhs.uk (E.Y.); robin.jones4@nhs.net (R.L.J.); spyridon.gennatas@nhs.net (S.G.); 4 Department of Medical Oncology, Netherlands Cancer Institute, 1066 CX Amsterdam, The Netherlands; 5 Department of Surgical Oncology, Netherlands Cancer Institute, 1066 CX Amsterdam, The Netherlands; w.v.houdt@nki.nl; 6 Department of Surgical Oncology, Radboud University Medical Center, 6525 GA Nijmegen, The Netherlands; han.bonenkamp@radboudumc.nl; 7 Division of Clinical Studies, Institute of Cancer Research, Royal Marsden NHS Foundation Trust, London SM2 5NG, UK; 8 Department of Medical Oncology, Guy’s and St Thomas’ NHS Foundation Trust, London SE1 9RT, UK

**Keywords:** desmoid-type fibromatosis, rare diseases, health-related quality of life, patient-reported outcomes, disease-specific measures

## Abstract

**Simple Summary:**

Desmoid-type fibromatosis (DTF) is an uncommon soft-tissue tumour that is incapable of metastasising. Patients can experience a variety of physical and psychological symptoms, making assessment of health-related quality of life (HRQoL) highly relevant. Measuring the impact of DTF on HRQoL can be challenging due to the rare character and variable clinical presentation of the disease. Therefore, a HRQoL instrument assessing DTF-specific issues is needed. Previously, a provisional DTF-specific HRQoL tool was developed (the DTF-QoL). The aim of the current study was to pre-test the DTF-QoL. Pre-testing of the questionnaire led to the selection of 96 questions, conceptualised into three symptom subscales, eleven disease-impact subscales and six single items, together forming the final DTF-QoL. This questionnaire can be used in both clinical and research settings to evaluate HRQoL of DTF patients, which could help to provide personalised care and improve overall patient experience.

**Abstract:**

Sporadic desmoid-type fibromatosis (DTF) is a rare, non-metastasising soft-tissue tumour. Patients can experience a variety of disease-specific issues related to the unpredictable clinical course and aggressiveness of DTF, which negatively impacts health-related quality of life (HRQoL). These DTF-specific issues are not captured by generic HRQoL tools. A 102-item provisional DTF-specific HRQoL tool, the DTF-QoL, was previously developed. The aim of this study was to pre-test the psychometric properties of the DTF-QoL by administering it together with the EORTC Quality of Life Questionnaire Core 30 (EORTC QLQ-C30) to 236 DTF patients from the United Kingdom and the Netherlands. Construct validity and reliability were determined based on factor analysis, multi-trait scaling analysis, Cronbach’s alpha, and correlations with the EORTC QLQ-C30 scales. Ninety-six items were selected, conceptualised into three symptom scales, eleven disease-impact scales and six single items, together forming the final DTF-QoL. Scaling assumptions were fully or moderately met for ten out of fourteen scales. Cronbach’s alpha ranged from 0.551–0.908. Most scales of the DTF-QoL were weakly or moderately correlated with the EORTC QLQ-C30. The DTF-QoL is a promising tool capturing the whole spectrum of DTF-specific issues. Implementation of the DTF-QoL in research and clinical practice will help to personalise HRQoL measurement and clinical care for DTF patients.

## 1. Introduction

Desmoid-type fibromatosis (DTF) is a rare soft-tissue tumour [1]. The estimated incidence in the population is 5–6 patients per million people per year, with a peak incidence between 20 and 40 years of age, and the majority of the patients being female [2,3]. Although DTF does not metastasise, its potential locally aggressive tumour growth can cause significant morbidity and it is therefore categorised as an intermediate tumour [1]. DTF tumours do occur in the context of familial adenomatous polyposis (FAP), but the majority of DTF tumours are sporadic and characterised by mutations in the β-catenin (*CTNNB1*) gene [4]. Sporadic DTF can arise in any part of the body, most commonly in the extremities and the abdominal wall [5]. The biological behaviour of DTF is unpredictable and variable, including phases of progressive growth, growth stabilisation, and spontaneous regression [6,7].

The unpredictable clinical course of DTF makes it challenging to choose an appropriate treatment strategy. Active surveillance is currently recommended as first-line treatment for asymptomatic and mildly symptomatic patients, while in the case of persistent progression or increasing symptoms, systemic therapies, surgical resection, and local therapies, such as radiotherapy, can be considered [4]. With high local recurrence rates for DTF at anatomic sites other than the abdominal wall, and with treatment-related toxicities, these active treatments do not guarantee tumour reduction or clinical benefit [4,5,8,9]. Potential risks and benefits of these treatment modalities should be considered carefully, as the main goal of treatment for DTF patients is to maintain acceptable health-related quality of life (HRQoL) [10].

HRQoL is a multidimensional concept that includes the patient’s perception of the impact of their disease and treatment on physical, psychological, and social functioning [11]. It provides additional information next to objective outcomes, such as radiological response or overall survival, to determine the net clinical benefit of a treatment. Integration of HRQoL assessment in clinical practice can improve patient satisfaction, communication and symptom control [12,13]; however, data on HRQoL in DTF patients are scarce. A recent study indicated that DTF patients generally have a similar or slightly worse HRQoL compared to the normative population [14]. Nevertheless, DTF patients do report a variety of disease-specific issues related to the unpredictable clinical course, rarity and aggressiveness of DTF, negatively impacting their HRQoL. Additionally, DTF patients can experience physical symptoms caused by the tumour itself or as a side effect of treatment [14,15,16]. These DTF-specific HRQoL issues are not captured by generic or cancer-generic HRQoL questionnaires, such as the European Organisation for Research and Treatment of Cancer Quality of Life Questionnaire Core 30 (EORTC QLQ-C30), which are predominantly used in DTF studies. The core EORTC QLQ-C30 consists of 30 items and was intended to be supplemented by disease-specific modules [17]. One DTF-specific tool is currently available, the ‘Gounder/DTRF Desmoid Symptom/Impact Scale’ (GODDESS), which has been developed according to the United States Food and Drug Administration (FDA) regulatory requirements for a disease-specific patient-reported outcome (PRO) instrument [18]. To develop a disease-specific tool for DTF patients that can be used to supplement the EORTC-QLQ-C30, we have developed a DTF-specific HRQoL questionnaire according to the guidelines of the EORTC Quality of Life Group [19]. Previously, interviews were organised and HRQoL issues were ranked according to their relevance by patients and healthcare providers to ensure content validity (phase I EORTC guidelines). Based on the results of these studies, the most important issues for DTF patients in several domains, including diagnosis, treatment, living with DTF, and healthcare facilities for DTF, were identified and transformed into 102 items of a provisional DTF-specific HRQoL tool, named the DTF-QoL (phase II EORTC guidelines). The aim of the current study is to pre-test the psychometric properties of the DTF-QoL (phase III EORTC guidelines).

## 2. Results

### Testing the Psychometric Properties of the Provisional DTF-QoL

The DTF-QoL and the EORTC QLQ-C30 were completed by 236 DTF patients (response rate 47%). Socio-demographic and clinical characteristics are described in Table 1.

Response information is provided in Table 2, which displays the items’ descriptive statistics. Valid responses were high for all items. Missing responses were considered as missing at random. Five items with >85% of the answers falling in the lowest category (floor effects) were excluded for factor analyses [holding a pen (item 11), lost weight (item 14), guilty receiving more attention than family members (item 44), suicidal thoughts (item 47), and changing living accommodation (item 54)]. Six items had high ceiling effects (80–85%); however, these items were considered to be of clinical importance for a subgroup of patients and were therefore retained. Factor analysis was performed for items 1-18 (time frame during the past week) and items 20–102 (time frame since diagnosis) separately. Item 19 (time frame last four weeks) was excluded for factor analyses due to a different time frame, and therefore seen as single item.

The suitability of the data (remaining 96 items) for factor analyses was tested via the Kaiser–Meyer–Olkin (KMO) measure of sampling adequacy, which tests the partial correlations among the items. The KMO value should be higher than 0.5 for a satisfactory analysis to proceed [20]. The KMO measures in this study were 0.89 (items 1–18) and 0.87 (items 20–102). Items with initial communalities <0.10 should be deleted for further analyses; none of the items were deleted based on this criterion. Based on Cattell’s scree plot and the Kaiser–Guttman rule (which states that an eigenvalue (the amount of the total variance explained by that factor) must be greater than one), three (items 1–18) and eleven factors (items 20–102) were identified. Factors for items with time frame ‘during the past week’ were numbered from W1 to W3, and factors for items with time frame ‘since your diagnosis’ from 1 to 11. Oblique rotation, which assumes that the factors could be correlated with each other, simplified their interpretation by minimising the items with high loadings on each factor. For the items with time frame ‘during the past week’, the three factors cumulatively accounted for 55% of variation in all items (respectively, 42%, 7%, 6%).

Factor analyses for items with time frame ‘since your diagnosis’ demonstrated eleven factors cumulatively accounting for 50% of variation in all items (respectively, 24%, 6%, 4%, 3%, 3%, 2%, 2%, 2%, 2%, 1%, 1%). Oblique rotation generates a pattern matrix with factor loadings (Table 3 and Table 4) and a structure matrix with correlations between items and components in a structure matrix. Cut-off points of 0.20 (items 1–18) and 0.15 (items 20–102) for factor loadings were adopted, i.e., only those items scoring higher than this threshold were retained for further analyses [21]. Based on this criterion, no items were dropped. Five items were treated as single items based on content validity (items 56, 67, 84, 89, and 95). Item 22 ‘Has the disease changed your life perspective (positive or negative)?’ was excluded due to incorrect wording and similarity to item 67 ‘Have you felt your desmoid fibromatosis has changed your life in a negative way?’

The multi-trait scaling analysis showed that scaling assumptions were fully met for three of the fourteen scales. Seven of the fourteen scales showed that scaling assumptions were moderately met, and four scales poorly met the scaling assumptions (Table 5). Cronbach’s alpha coefficients ranged from 0.551–0.908, and ten of the fourteen scales scored higher than the preferred 0.70 level. Removing items improved the Cronbach’s alpha coefficient of five scales. In Table S1, these items were distributed into the scales with which they had the highest correlation to assess whether this resulted in an improved scale structure and higher scale reliability, except for item 46. Removing item 46 ‘Have you felt supported by your family members and/or friends?’ from scale 4 (‘effect of DTF on relationships’) did improve the Cronbach’s alpha coefficient of scale 4 (from 0.908 to 0.920). However, based on the content validity of item 46 with scale 4, no higher correlation of item 46 with another scale, and only little improvement of Cronbach’s alpha, item 46 was not distributed to another scale.

Scales of the DTF-specific questionnaire measuring similar concepts as scales of the EORTC QLQ-C30 showed high correlations. For example, scale W2 (‘physical consequences’) and scale 5 (‘physical limitations and consequences’) of the DTF-QoL were strongly correlated with the physical functioning scale of the EORTC QLQ-C30 (Table 6). Most scales of the DTF-QoL were weakly or moderately correlated (*r* ≤ 0.60) with the EORTC QLQ-C30 (Table 6).

The final version of the DTF-QoL consisted of 96 items (Supplementary Questionnaire).

## 3. Discussion

The DTF-QoL has been developed according to the robust guidelines of the EORTC Quality of Life Group to measure disease-specific and treatment-related HRQoL issues relevant to DTF patients that are not adequately covered by the EORTC QLQ-C30 [19]. The DTF-QoL is designed to be used in combination with the EORTC QLQ-C30. The content of the questionnaire has been determined by an extensive literature search, and by interviews with healthcare providers and DTF patients from two countries [14,16]. This made it possible to pre-test the questionnaire in a relatively large study sample for a rare disease and to enhance cross-cultural acceptability.

Exploratory factor analysis of the DTF-QoL yielded fourteen factors, which were categorised in three symptom scales (items with time frame during the past week) and eleven disease impact scales (items with time frame since diagnosis). These scales covered all the DTF-specific issues found in previous studies [14,15,16]. Results confirmed the hypothesised scale structure for three of the fourteen scales. Specifically, high reliability estimates (0.804–0.893) and scaling success were achieved for scale 1 (‘concerns about condition’), 2 (‘job and education’) and W3 (‘pain and discomfort’). However, some limitations should be acknowledged. Although reliability and item–scale convergent validity were satisfactory for scale W1 (‘emotional and psychological consequences’), W2 (‘psychological consequences’), 4 (‘effect of DTF on relationships’), 5 (‘physical limitations and consequences’), 8 (‘body image and sensation’), and 11 (‘unpredictable course and nature of DTF’), item–scale discriminant validity was suboptimal. The fact that some items showed a higher correlation with other scales may be due to common themes the scales contain. Lower reliability and poor item–scale convergent and divergent validity were observed for scale 6 (‘diagnostic and treatment trajectory of DTF’), 7 (‘parents and fertility’), 9 (‘supportive care’) and 10 (‘concerns around treatment and its consequences’). Despite the suboptimal results of some scales, we prefer to keep the scale structure of all scales, as scales improve clinical interpretability [15].

Based on the content validity of an item with its scale, or improvement of the Cronbach’s alpha coefficient if a certain item was deleted, we explored whether rearrangement of some items would improve the psychometric properties of the scales. For scale 3 (‘doctor–patient relationship, communication and information’), item 78 ‘Have you experienced a lack of continuity (seeing the same doctors / specialised nurse) in the care for your desmoid fibromatosis?’ showed a weak correlation with its hypothesised scale, but had no higher correlation with another scale. In addition, removing item 46 ‘Have you felt supported by your family members and/or friends?’ from scale 4 (‘effect of DTF on relationships’) improved the reliability of scale 4. The fact that item 46 might be more person-specific rather than DTF-specific might explain why this item did not fit scale 4. Furthermore, both items 78 and 46 reflected a more generic aspect related to support, which was associated with almost all scales. For this reason, treating item 46 and 78 as single items could be considered. Moving item 52 ‘Have you felt a change in sensation in the area around the tumour?’ from scale 8 (‘body image and sensation’) to 10 (‘concerns around treatment and its consequences’) improved the Cronbach’s alpha coefficients, as well as the scaling fulfilment of both scales. Based on these results and good content validity, we recommend to move item 52 from scale 8 to scale 10. Finally, item 68 ‘Have you wanted to meet others with desmoid fibromatosis?’ of scale 9 (‘supportive care’) was formulated in a neutral way, so patients could have interpreted it positively or negatively, possibly affecting the reliability of the scale. The fact that Cronbach’s alpha was negative reflected the incoherence of item 68 with its scale. We chose not to consider the items of scale 9 as a single item directly, but suggest formulating item 68 in a positive direction and re-evaluating the psychometric properties of this scale. However, all our recommendations, as well as our hypothesised scale structure, require additional confirmatory analysis in a larger (international) study.

Up until now, one disease-specific HRQoL instrument, the GODDESS, has been developed for DTF patients [18]. The GODDESS, consisting of 28 items, enhanced the possibility of assessing DTF-specific HRQoL. Our DTF-specific HRQoL questionnaire can be used in conjunction with the cancer-generic EORTC QLQ-C30 questionnaire, for better comparison with cancer patient populations potentially receiving similar treatments and with normative populations. The time frames of the DTF-QoL (one week for symptom items; since diagnosis for impact items) are longer than those of the GODDESS tool (24 h for symptom items; one week for impact items) to ensure that important symptom-related information can be captured at less frequent time points. The study to develop the GODDESS included patients with FAP-related DTF; in our current study FAP-patients were excluded. FAP-related DTF seems to be characterised by a more aggressive clinical course, and subsequently a different treatment strategy, which can lead to different HRQoL compared to patients with sporadic DTF [10,22]. We hypothesise that the EORTC QLQ-C30 can also be supplemented with the DTF-QoL in FAP-patients to evaluate their DTF-specific HRQoL. However, since FAP-patients were excluded in our study, the use of the DTF-QoL in FAP-patients must be further studied.

Finally, the DTF-QoL consists of a relatively large number of items compared to the GODDESS, and may be exhausting to patients. However, all items were considered to be of clinical relevance, as these issues were rated as important by patients and healthcare providers during phase I of the development process. In particular, the impact scales of the DTF-QoL include a variety of DTF-specific HRQoL issues not covered by existing questionnaires, such as the unpredictable disease trajectory of DTF or the effect of DTF on relationships. The weak correlation of most scales with the EORTC QLQ-C30 confirms divergent validity and supports the fact that the DTF-QoL identifies issues not covered by generic or cancer-generic questionnaires. Known-group comparisons will be performed next to assess the ability of the DTF-QoL to discriminate between subgroups of DTF patients with different disease phase or treatment modalities. Furthermore, based on the results of these subgroup analyses, relevant and irrelevant issues for specific subgroups will be identified. Ultimately, we hope to identify optional and essential scales for specific subgroups or even for individual patients. Furthermore, the use of different time frames offers the possibility of using a specific selection of subscales for a specific type of research. For example, the symptom scales with a relatively short time frame (past week) are appropriate for assessing a patient’s experience during a clinical trial, while the disease impact scales can potentially be used during longitudinal follow-up. Given the large number of items, which together form an extensive item library, it might be worthwhile to explore the possibilities of applying computer adaptive testing (CAT) techniques [23]. CAT techniques will help to evaluate HRQoL more precisely with fewer items, which will personalise the DTF HRQoL scores even more.

## 4. Materials and Methods

### 4.1. Questionnaire Development

To develop the DTF-QoL, the EORTC guidelines for developing questionnaire modules were followed as much as possible, however our questionnaire is not an EORTC product [19]. The development consisted of three distinct phases. Phase I and II have been conducted previously, and are described in detail elsewhere [16,24]. The provisional DTF-specific HRQoL questionnaire, the DTF-QoL, was pre-tested in a sample of DTF patients to test construct validity and reliability.

### 4.2. Study Sample and Data Collection

The sample included 236 patients (United Kingdom (UK): *n* = 80; Netherlands (NL): *n* = 156) who participated in the QUALIFIED study (The evaluation of health-related quality of life issues experienced by patients with desmoid-type fibromatosis; registered at clinicaltrials.gov (accessed on 1 July 2021): NCT04289077) [24]. The QUALIFIED study is an international, multicentre, cross-sectional, observational cohort study among adult (≥18 years) patients with sporadic DTF who were treated in one of the participating centres (one centre in the UK; three centres in the NL). Patients completed a set of questionnaires including the EORTC QLQ-C30 and DTF-QoL. Questionnaire data were collected via the PROFILES registry and management system; an established international registry for collection of cancer patient reported outcomes [25]. Sociodemographic and clinical data were extracted from the questionnaire (patient-reported) and from the patient medical records. Ethical approval was obtained in each participating centre in the UK and the NL (Royal Marsden Hospital: SE806, Erasmus Medical Centre: MEC-2019-0816, Radboudumc: file number 2020-6235, Netherlands Cancer Institute: IRBd20-088). Further details of the protocol are described elsewhere [24].

### 4.3. Statistical Analyses

Patient characteristics were summarised using descriptive statistics. For phase III, item descriptive statistics and response distributions for each item were calculated, in order to examine central tendency, variability and symmetry. Floor or ceiling effects were considered high if >85% of answers fell in the lowest or highest category respectively [26].

To support construct validity, exploratory factor analysis was used to determine underlying constructs that explained significant portions of the variance. The factor loadings, i.e., the correlation coefficients between the items and the factors, were examined in order to explain the meaning of each construct. Items deemed inappropriate for the identified scales due to their content were treated as single items. Scores of the identified scales were calculated according to the guidelines of the EORTC Quality of life group [27].

Multi-trait scaling analysis was performed to confirm the hypothesised scale structure of the questionnaire [28]. To test for item–scale convergent validity, the correlation between each individual item and its own scale (corrected for overlap) was examined. Correlations of ≥0.40 were considered substantial and satisfactory. By comparing the correlation of each item with its own scale versus other scales, item–scale discriminant validity was examined. It was expected that an item correlated higher with its hypothesised scale compared to other scales.

Reliability was assessed by calculating Cronbach’s alpha coefficient (ranges between 0 and 1). A minimum score of 0.70 is preferred [29]. Substantially lower scores indicate an unreliable scale. Cronbach’s alpha was also calculated for each scale per item if that item were deleted, to see if the scale improved without the item.

Finally, Pearson’s correlation coefficients were calculated between the scales of the EORTC QLQ-C30 and the identified scales of the DTF-QOL to assess convergent and divergent validity. Scales conceptually related were sought to show moderate to high correlations (*r* ≥ 0.40), while scales with less conceptual relation were expected to correlate weakly (*r* < 0.40) [29].

Missing answers for patients completing the questionnaires were reported where applicable, and only available data were used for further analyses. All analyses were performed using SPSS software, version 25.0 (SPSS Inc., Chicago, IL, USA).

## 5. Conclusions

The DTF-QoL is the first DTF-specific questionnaire developed using the EORTC guidelines for developing a questionnaire. This questionnaire can be a useful HRQoL instrument in future (longitudinal) clinical studies and clinical care. It could help healthcare professionals to understand patient priorities and to recognise HRQoL issues earlier to provide truly personalised care and improve overall patient experience.

## Figures and Tables

**Table 1:** Desmoid-type fibromatosis patient characteristics (*n* = 236).

		Mean (SD)
Age in years at time of diagnosis		41.6 (14.4)
Age in years at time of questionnaire		47.2 (14.0)
		*n* (%)
Sex	Male	62 (26.3)
	Female	174 (73.7)
Tumour localisation	Head/neck	13 (5.5)
	Upper extremity/shoulder	29 (12.3)
	Trunk ^1^	54 (22.9)
	Abdominal wall	58 (24.6)
	Intra-abdominal	39 (16.5)
	Hip/pelvis/gluteal region	21 (8.9)
	Lower extremity	22 (9.3)
Recurrent disease after surgery (*n* = 98)	Yes	41 (41.8)
	No	57 (58.2)
Treatment received ^2^	Active surveillance only	87 (36.9)
	Any form of active treatment	149 (63.1)
Comorbidity (self-report)	None	91 (38.6)
	≥1	145 (61.4)
Relationship status	Partnered	182 (77.1)
	Not partnered	53 (22.5)
	Missing	1 (0.4)
Education level	Low (primary/secondary)	36 (15.3)
	Medium (vocation/college/diploma)	127 (53.8)
	High (university/post-graduate)	73 (30.9)
Current employment status	Working	156 (66.1)
	Not working	80 (33.9)

^1^. Including thoracic wall, breast, and back. ^2^. Active surveillance only: including patients who received only active surveillance or analgesics; Active treatment: including patients who received surgery, systemic therapy or targeted therapy (i.e., radiotherapy, isolated limb perfusion, high-intensity focused ultrasound, cryoablation).

**Table 2:** Item descriptive statistics of 236 desmoid-type fibromatosis patients.

Item	Description	Valid (%)	Mean (SD)	Distribution of Valid Responses (%)				
				1	2	3	4	NA
	During the Past Week:							
1	Have you felt uncertain?	100	1.6 (0.8)	55.9	31.4	8.9	3.8	-
2	Has pain interfered with your sleep at night?	99.6	1.5 (0.8)	67.7	19.6	8.1	4.7	-
3	Have you had a bad temper because of the condition?	100	1.4 (0.6)	71.2	21.6	6.4	0.8	-
4	Have you been unsatisfied with your body?	100	1.7 (0.9)	52.5	28.8	12.3	6.4	-
5	Have you had problems getting dressed?	100	1.2 (0.5)	84.3	13.1	2.1	0.4	-
6	Have you felt isolated?	100	1.3 (0.6)	80.5	14.4	2.5	2.5	-
7	Have you felt disabled?	100	1.3 (0.6)	78.0	17.4	2.5	2.1	-
8	Have you had pain while sitting?	100	1.5 (0.8)	64.8	22.9	7.2	5.1	-
9	Have you had stiffness in your limbs?	100	1.6 (0.8)	57.2	29.2	8.9	4.7	-
10	Have you had any trouble walking?	99.6	1.4 (0.7)	71.9	21.3	4.7	2.1	-
11	Did you have a problem holding a pen, which made writing difficult? *	100	1.1 (0.5)	91.5	5.1	2.5	0.8	-
12	Have you had swelling in your legs or ankles (oedema)?	100	1.3 (0.7)	81.4	12.3	3.4	3.0	-
13	Have you been unable to lean on the tumour sites? (e.g., due to local pressure)?	100	1.7 (1.0)	59.3	19.1	11.0	10.6	-
14	Have you lost weight? *	100	1.2 (0.5)	87.3	10.2	1.7	0.8	-
15	Have you felt lonely?	100	1.3 (0.7)	76.3	16.9	4.7	2.1	-
16	Have you worried about the disease being aggressive?	100	1.6 (0.9)	60.2	25.4	7.6	6.8	-
17	Have you worried about dying?	100	1.4 (0.7)	75.4	17.8	2.1	4.7	-
18	Have you had problems with your appearance?	99.6	1.4 (0.8)	71.5	20.4	3.0	5.1	-
	In the last four weeks:							
19	Have you had a decreased libido?	100	1.8 (1.0)	50.4	14.8	15.7	7.2	11.9
	Since your diagnosis:							
20	Have you been afraid of tumour growth?	100	2.5 (0.9)	12.3	45.8	25.0	16.9	-
21	Have you felt there is something in your body that does not belong there?	99.6	2.6 (1.1)	17.0	36.2	18.7	28.1	-
22	Has the disease changed your life perspective (positive or negative)?	99.6	2.3 (1.0)	25.5	33.6	23.8	17.0	-
23	Has desmoid fibromatosis or its treatment caused those close to you to feel distressed?	99.6	2.3 (1.0)	24.3	35.7	26.4	13.6	-
24	Have you been afraid of getting another tumour?	99.6	2.2 (1.0)	31.9	36.6	15.7	15.7	-
25	Have you been worried or concerned about the future?	99.6	2.3 (1.0)	21.3	39.6	23.4	15.7	-
26	Have you felt a need to keep your fears, concerns and/or symptoms from family members or friends?	99.6	2.0 (1.1)	44.3	25.5	18.3	11.9	-
27	Has your physical fitness level reduced?	99.6	2.0 (1.0)	40.4	29.8	19.1	10.6	-
28	Have you felt asymmetrical and/or misshapen due to the desmoid fibromatosis or the treatment?	99.6	1.9 (1.0)	46.8	28.9	9.8	12.8	1.7
29	Have you felt worried constantly?	99.6	1.9 (0.9)	39.6	39.1	16.2	5.1	-
30	Have you been disappointed by the course of your condition?	99.6	1.8 (0.9)	51.1	27.2	14.9	6.8	-
31	Have you felt you had to fight this condition?	99.6	1.8 (1.0)	51.9	23.0	14.9	10.2	-
32	Have you had problems with confidence?	99.6	1.7 (0.9)	55.3	26.4	14.0	4.3	-
33	Have you worried that you are a burden to other people?	99.6	1.8 (0.9)	51.5	29.4	11.1	8.1	-
34	Have you had difficulties explaining your condition to others?	99.6	1.9 (1.0)	48.9	28.1	11.5	11.5	-
35	Do you think your condition is not well understood by people close to you?	99.6	1.8 (1.0)	55.3	23.4	12.3	8.9	-
36	Have you felt less independent and/or more dependent on others?	99.6	1.5 (0.8)	66.8	20.9	7.7	4.7	-
37	Have you had problems eating?	99.6	1.2 (0.6)	84.3	11.1	2.1	2.6	-
38	Has the desmoid fibromatosis had a negative impact on your family life?	99.6	1.6 (0.8)	59.6	25.5	10.6	4.3	-
39	Has your physical condition or medical treatment interfered with your social activities?	100	1.8 (0.9)	47.9	28.4	17.8	5.9	-
40	Have you had extra expenses due to your physical condition or medical treatment (e.g., for medication, transport and/or aids)?	100	1.8 (0.9)	44.5	38.6	10.2	6.8	-
41	Has your physical condition or medical treatment interfered with your marriage or intimate relationships?	99.6	1.7 (1.0)	54.0	20.9	10.6	7.7	6.8
42	Have you had problems with your ability to have children because of your desmoid fibromatosis?	99.6	1.4 (0.9)	50.6	3.4	2.6	4.7	38.7
43	Have you felt that you have received less attention from family and friends because the condition is benign?	100	1.4 (0.9)	75.0	12.7	5.9	6.4	-
44	Have you felt guilty for receiving more attention than family members (e.g., siblings) because of your desmoid fibromatosis? *	99.6	1.2 (0.5)	88.5	8.9	1.7	0.9	-
45	Have you had problems with your job or your education?	99.6	1.8 (1.1)	51.1	15.7	10.6	10.6	11.9
46	Have you felt supported by your family members and/or friends?	100	3.3 (0.9)	6.4	13.6	26.7	53.4	-
47	Have you had suicidal thoughts? *	100	1.2 (0.5)	88.6	8.9	1.3	1.3	-
48	Have you had problems driving a car?	99.6	1.4 (0.7)	70.2	22.6	5.5	1.7	-
49	Have you lost friendships?	99.2	1.3 (0.7)	82.5	11.1	3.0	3.4	-
50	Have you been afraid of needing a limb amputation?	99.6	1.5 (0.9)	58.3	11.9	4.3	6.8	18.7
51	Have you been afraid of your desmoid fibromatosis coming back?	99.6	2.5 (1.0)	12.3	31.9	19.1	18.7	17.9
52	Have you felt a change in sensation in the area around the tumour?	99.6	2.4 (1.1)	26.4	31.9	19.6	22.1	-
53	Have you wanted to cover-up the tumour area and /or scar(s)?	100	1.8 (1.1)	59.3	13.1	12.7	14.8	-
54	Have you changed your living accommodation because of desmoid fibromatosis? *	100	1.2 (0.6)	92.4	3.0	1.7	3.0	-
55	Have you felt addicted to pain medication?	100	1.2 (0.6)	78.0	8.1	1.7	3.0	9.3
56	Have you lost your hair?	100	1.3 (0.7)	79.7	11.4	5.5	3.4	-
57	Has your parental role been affected because of your desmoid fibromatosis?	100	1.4 (0.8)	54.2	11.4	3.4	3.0	28.0
58	Have you felt that doctors are unfamiliar with desmoid-type fibromatosis?	100	2.3 (1.0)	27.5	32.2	24.6	15.7	-
59	Have you felt stressed around check-ups?	100	2.2 (0.9)	20.8	46.2	22.0	11.0	-
60	Have you felt frustrated about the ’benign’ diagnosis with cancerous features?	99.6	2.1 (1.1)	39.6	27.2	18.7	14.5	-
61	Have you felt stressed about the diagnosis?	99.6	2.5 (0.9)	13.2	40.0	30.2	16.6	-
62	Did you think it took a long time to get a definite diagnosis?	100	2.1 (1.1)	43.6	23.3	16.1	16.9	-
63	Have you felt like a cancer patient?	100	2.0 (0.9)	33.5	39.4	18.2	8.9	-
64	Have you felt reassured by the benign nature of your disease?	100	2.6 (1.0)	14.8	35.2	25.0	25.0	-
65	Do you think your prognosis (the expected improvement or worsening of your condition) is clear?	100	2.3 (1.0)	30.5	25.0	30.9	13.6	-
66	Have you felt there is no optimal treatment for you?	100	2.2 (1.1)	33.9	29.7	19.9	16.5	-
67	Have you felt your desmoid fibromatosis has changed your life in a negative way?	100	2.0 (1.0)	37.7	35.2	15.7	11.4	-
68	Have you wanted to meet others with desmoid fibromatosis?	99.6	1.8 (1.0)	49.8	30.6	9.4	10.2	
69	Have you had doubts about the effectiveness of your treatments?	100	1.9 (1.0)	38.6	28.4	13.6	9.7	9.7
70	Have you found it frustrating having to explain your condition to others?	100	1.8 (1.0)	50.0	29.7	12.3	8.1	-
71	Have you lacked information about your desmoid fibromatosis and/or its treatment?	100	1.6 (0.8)	60.2	25.4	9.7	4.7	-
72	Have you been satisfied with your communication with your professional(s)?	99.6	3.1 (0.9)	7.7	14.5	34.9	43.0	-
73	Have you had to explain your circumstances to others?	100	2.0 (0.9)	36.0	36.4	19.1	8.5	-
74	Do you feel like you have a chronic disease?	100	2.1 (1.1)	37.7	31.4	16.9	14.0	-
75	Have you lacked psychological support?	100	1.6 (0.9)	63.6	20.8	11.0	4.7	-
76	Have you had to take sick leave?	99.6	2.2 (1.2)	33.6	17.9	15.7	17.4	15.3
77	Have your career ambitions changed because of the diagnosis?	99.2	1.8 (1.1)	52.1	14.1	12.4	11.5	9.8
78	Have you experienced a lack of continuity (seeing the same doctors / specialised nurse) in the care for your desmoid fibromatosis?	100	1.7 (0.9)	58.9	22.0	11.9	7.2	-
79	Have you had problems receiving enough information about your desmoid fibromatosis and its treatment?	100	1.6 (0.8)	57.6	29.7	8.1	4.7	-
80	Have you been bothered by long travel to the hospital?	100	1.6 (0.8)	55.9	30.9	8.9	4.2	-
81	Have you worried about passing the condition to your children?	100	1.7 (1.0)	46.6	20.8	7.6	8.5	16.5
82	Have you had a problem receiving treatments in a cancer hospital?	100	1.5 (0.8)	64.8	24.2	8.1	3.0	-
83	Have you worried about not being able to continue working or studying?	99.6	2.0 (1.1)	40.0	20.0	10.6	14.5	14.9
84	Has the colour of your hair changed?	99.2	1.2 (0.6)	78.2	8.1	2.6	1.7	9.4
85	Have you felt that you are standing still in life?	100	1.8 (1.0)	55.9	19.5	13.6	11.0	-
86	Have you felt less feminine/masculine?	100	1.4 (0.8)	77.5	12.3	5.1	5.1	-
87	Have you received different contradictory recommendations about treatment options?	100	1.5 (0.8)	72.0	14.0	9.7	4.2	-
88	Have you had to change jobs as a result of your condition?	99.6	1.5 (1.0)	62.1	3.8	3.8	8.5	21.7
89	Have you felt you were wasting the time of cancer specialists?	100	1.4 (0.7)	75.0	17.8	4.7	2.5	-
90	Have you worried about your ability to have children?	99.6	1.7 (1.1)	38.3	9.4	2.6	9.4	40.4
91	Were you frightened by the referral to the cancer hospital?	100	2.1 (1.1)	38.6	27.1	20.3	14.0	-
92	Have you lacked online support (forum and/or chat group)?	99.6	1.5 (0.9)	69.4	18.7	6.0	6.0	-
93	Has there been mutual trust between you and your professional(s)?	100	3.3 (0.8)	3.0	9.3	39.0	48.7	-
94	Have you felt embarrassed using mobility aids (e.g., wheelchair, scooter, electric bike)?	99.2	1.5 (0.9)	24.8	5.6	1.7	2.6	65.4
95	Have you had a rash as a result of the treatment?	99.2	1.4 (0.8)	79.1	11.5	3.8	5.6	-
96	Have you worried about your treatment?	99.6	2.1 (1.0)	34.0	36.6	17.0	12.3	-
97	Have you worried about a decrease in muscle strength after treatment?	99.6	1.9 (1.1)	52.8	16.6	16.2	14.5	-
98	Have you received enough information about the possible treatment side effects?	99.2	2.6 (1.1)	24.8	16.7	35.5	23.1	-
99	Have you been worried about tumour growth during pregnancy?	99.2	2.4 (1.3)	15.0	4.3	3.4	12.0	65.4
100	Were you passed from one hospital to another before the final desmoid fibromatosis diagnosis?	100	1.9 (1.1)	51.7	25.0	6.4	16.9	-
101	Have you worried about being treated unfairly because of your desmoid fibromatosis (i.e., at work, by insurance companies)?	100	1.6 (0.9)	65.3	18.2	9.3	7.2	-
102	Have you worried about being able to access treatments in the future?	98.7	1.6 (0.9)	59.2	22.7	9.9	6.0	2.1

Valid (%) is the percentage of patients who answered the question. 1, Not at all; 2, A little; 3, Quite a bit; 4, Very much; NA, not applicable; * Items 11, 14, 44, 47, and 54 were deleted due to floor effects.

**Table 3:** Rotated component matrix (factor loadings pattern matrix) items with time frame during the past week.

Item	Description	Pattern Matrix		
		1	2	3
Factor W1: Emotional and psychological consequences				
4	Unsatisfied with body	0.782		
18	Problems with appearance	0.748		
6	Felt isolated	0.735		
15	Felt lonely	0.653		
17	Worried about dying	0.646		
1	Felt uncertain	0.628		
16	Worried about aggressive disease	0.485		
3	Bad temper because of condition	0.241		
Factor W2: Physical consequences				
10	Trouble walking		0.772	
12	Swelling leg/ankles		0.657	
7	Felt disabled		0.338	
9	Stiffness in limbs		0.296	
5	Problems getting dressed		0.227	
Factor W3: Pain and discomfort				
2	Pain interfered with sleep			−0.910
8	Pain while sitting			−0.714
13	Unable to lean on tumour site			−0.694

**Table 4:** Rotated component matrix (factor loadings pattern matrix) items with time frame since diagnosis.

Item	Description	Pattern Matrix										
		1	2	3	4	5	6	7	8	9	10	11
	Factor 1: Concerns about condition	1										
20	Afraid tumour growth	0.738										
24	Afraid getting another tumour	0.636										
25	Worried about future	0.626										
29	Worried constantly	0.601										
61	Stressed about diagnosis	0.581										
96	Worried about treatment	0.535										
23	People close feel distressed	0.508										
59	Stressed check-ups	0.438										
	Factor 2: Job and education		2									
76	Sick leave		0.876									
45	Problems with job or education		0.867									
83	Worried not being able to work/study		0.845									
77	Career ambitions changed		0.741									
88	Change jobs		0.738									
	Factor 3: Doctor–patient relationship, communication and information			3								
93	Mutual trust between you and professional(s)			−0.689								
72	Satisfied communication professional(s)			−0.644								
98	Enough information treatment side effects			−0.596								
79	Problems receiving enough information DTF			0.569								
71	Lacked information DTF			0.431								
78	Lack of continuity in care for DTF			0.355								
	Factor 4: Effect of DTF on relationships				4							
43	Less attention family and friends because benign condition				0.643							
35	Condition not well understood by people				0.629							
34	Difficulties explaining to others				0.612							
32	Problems with confidence				0.423							
70	Frustrating explain condition to others				0.419							
46	Supported by family/friends				−0.409							
73	Explain circumstances to others				0.352							
26	Need to keep fears/concerns/symptoms from family/friends				0.337							
38	DTF negative impact on family				0.302							
33	Worried burden to other people				0.293							
101	Worried being treated unfairly				0.208							
41	Condition interfered relationships				0.178							
85	Felt standing still in life				0.159							
	Factor 5: Physical limitations and consequences					5						
36	Less independent/more dependent on others					−0.726						
40	Extra expenses due to condition or treatment					−0.653						
39	Condition interfered with social activities					−0.555						
27	Physical fitness reduced					−0.532						
48	Problems driving a car					−0.510						
49	Lost friendships					−0.402						
37	Problems eating					−0.317						
94	Embarrassed using mobility aids					−0.305						
	Factor 6: Diagnostic and treatment trajectory of DTF						6					
82	Problems treatments in cancer hospital						0.557					
100	Passed from one hospital to another before final diagnosis						0.544					
62	Long time to definite diagnosis						0.488					
58	Felt doctors unfamiliar with DFT						0.398					
87	Received contradictory recommendations treatment						0.295					
80	Bothered by long travel to hospital						0.234					
91	Frightened by referral to cancer hospital						0.198					
	Factor 7: Parents and fertility							7				
99	Worried tumour growth during pregnancy							−0.661				
42	Problems ability to have children because of DTF							−0.600				
57	Parental role affected because of DTF							−0.521				
90	Worried about ability to have children							−0.488				
81	Worried about passing condition to children							−0.451				
	Factor 8: Body image and sensation								8			
53	Cover−up tumour /scars								−0.469			
52	Change in sensation area tumour								−0.269			
86	Less feminine/masculine								−0.243			
28	Asymmetrical/misshapen due to DTF or treatment								−0.187			
	Factor 9: Supportive care									9		
92	Lacked online support									0.552		
68	Wanted to meet others									0.321		
75	Lacked psychological support									0.264		
	Factor 10: Concerns around treatment and its consequences										10	
50	Afraid needing limb amputation										−0.469	
55	Felt addiction to pain medication										−0.409	
69	Doubts effectiveness treatment										−0.327	
97	Worried decrease muscle strength after treatment										−0.233	
	Factor 11: Unpredictable course and nature of DTF											11
66	No optimal treatment											−0.574
65	Prognosis is clear											0.465
30	Disappointed by course of condition											−0.363
74	Feel like chronic disease											−0.331
31	Felt to fight condition											−0.293
21	Something in body that does not belong there											−0.233
64	Reassured by benign nature disease											0.210
102	Worried access treatments in future											−0.197
60	Frustrated benign diagnosis with cancerous features											−0.175
51	Afraid DTF coming back											0.173
65	Felt like cancer patient											−0.158

Item 22 (disease changed life perspective positive or negative) was excluded due to incorrect wording; Based on content validity items 56 (lost hair), 67 (DTF changed life negative way), 84 (colour hair changed), 89 (felt wasting time cancer specialists), 95 (rash result treatment) were seen as single items. Abbreviations: DTF, desmoid-type fibromatosis.

**Table 5:** Summary of results of multi-trait scaling.

					Item–Scale Convergent Validity Criterion 1 (Inclusive Criterion)		Item–Scale Divergent Validity Criterion 2 (Exclusive Criterion)		Scaling Fulfilment
Scale		*n*	Mean (SD) ^1^	Cronbach’s Alpha	Range of Item–Scale Correlations ^2^	Number of Item–Scale Correlations ^3^	Range of Correlations with Other Scales ^4^	Number of Items Higher Correlation with Other Scale ^5^	Number of Items that Meet Criterion 1 but not 2
	Time frame: during the past Week								
W1	Emotional and psychological consequences	8	15.4 (18.6)	0.875	0.552–0.712	8/8	0.333–0.611	1/8	7/8
	(Item 4; 18; 6; 15; 1; 17; 16; 3)								
W2	Psychological consequences	5	11.6 (16.5)	0.803	0.465–0.737	5/5	0.186–0.665	2/5	3/5
	(Item 10; 12; 7; 9; 5)								
W3	Pain and discomfort	3	19.5 (25.5)	0.804	0.623–0.708	3/3	0.431–0.585	0/3	3/3
	(Item 2; 8; 13)								
	Time frame: since diagnosis								
1	Concerns about condition	8	41.3 (24.1)	0.893	0.560–0.800	8/8	0.028–719	0/8	8/8
	(Item 20; 24; 25; 29; 61; 96; 23; 59)								
2	Job and education	5	29.1 (31.5)	0.887	0.699–0.895	5/5	0.026–0.729	0/5	5/5
	(Item 76; 45; 83; 77; 88)								
3	Doctor–patient relationship, communication and information	6	26.8 (19.6)	0.728	0.334–0.598	5/6	0.001–0.398	0/6	5/6
	(Item 93; 72; 98; 79; 71; 78)								
4	Effect of DTF on relationships	13	24.8 (21.8)	0.908 *	0.121–0.778	12/13	0.004–0.709	4/13	8/13
	(Item 43; 35; 34; 32; 70; 46; 26; 38; 101; 73; 33; 41; 85)								
5	Physical limitations and consequences	8	18.8 (21.8)	0.868 *	0.459–0.768	8/8	0.014–0.731	2/8	6/8
	(Item 36; 40; 39; 27; 48; 49; 37; 94)								
6	Diagnostic and treatment trajectory of DTF	7	28.1 (19.1)	0.680 *	0.261–0.550	3/7	0.053–0.489	3/7	3/7
	(Item 80; 82; 100; 62; 58; 87; 91)								
7	Parents and fertility	5	21.1 (19.1)	0.615	0.271–0.482	2/5	0.014–0.733	3/5	1/5
	(Item 99; 42; 57; 90; 81)								
8	Body image and sensation	4	28.9 (23.1)	0.755 *	0.446–0.648	4/4	0.001–0.711	2/4	2/4
	(Item 52; 53; 86; 28)								
9	Supportive care	3	36.1 (14.1)	−0.822 *	0.075–0.507	1/3	0.076–0.614	3/3	0/3
	(Item 92; 68; 75)								
10	Concerns around treatment and its consequences	4	22.0 (21.4)	0.551	0.294–0.434	1/4	0.003–0.621	4/4	0/4
	(Item 50; 55; 69; 97)								
11	Unpredictable course and nature of DTF	11	28.8 (22.4)	0.866	0.400–0.688	11/11	0.051–0.742	3/11	8/11
	(Item 66; 65; 30; 74; 31; 21; 64; 102; 60; 51; 63)								

Scaling assumptions were fully met if all items met criterion 1 but not 2; scaling assumptions were moderately met if ≥50% of the items met criterion 1 but not 2; scaling assumptions were poorly met if <50% met criterion 1 and not 2. * Cronbach’s alpha would improve if one of the items were deleted. ^1^ Higher scores indicate a higher level of symptomatology/problems. ^2^ Pearson correlation between items and hypothesised scale (corrected for overlap). ^3^ Number of item–scale correlations that meet minimum standard for convergent validity. ^4^ Pearson correlations between items and other scales. ^5^ Correlations higher between items and other scales in comparison with hypothesised scale. Abbreviations: DTF, desmoid-type fibromatosis

**Table 6:** Correlations between EORTC QLQ-C30 and scales DTF-QoL.

EORTC QLQ−C30	Factor W1	Factor W2	Factor W3	Factor 1	Factor 2	Factor 3	Factor 4	Factor 5	Factor 6	Factor 7	Factor 8	Factor 9	Factor 10	Factor 11
Physical functioning	−0.49 **	−0.76 **	0.58 **	−0.21 **	−0.55 *	−0.11	−0.39 **	−0.61 **	−0.20 **	−0.47 **	−0.39 **	−0.07	−0.36 **	−0.35 **
Role functioning	−0.59 **	−0.62 **	−0.65 **	−0.33 **	−0.60 **	−0.08	−0.46 **	−0.68 **	−0.25 **	−0.42 **	−0.44 **	0.038	−0.39 **	−0.43 **
Emotional functioning	−0.66 **	−0.54 **	−0.48 **	−0.47 **	−0.54 **	−0.16 *	−0.60 **	−0.59 **	−0.42 **	−0.41 **	−0.42 **	−0.15 *	−0.50 **	−0.54 **
Cognitive functioning	−0.52 **	−0.52 **	−0.40 **	−0.31 **	−0.50 **	−0.17 **	−0.43 **	−0.53 **	−0.22 **	−0.23 **	−0.22 **	−0.18 **	−0.39 **	−0.42 **
Social functioning	−0.67 **	−0.66 **	0.58 **	−0.40 **	−0.58 **	−0.14 *	−0.56 **	−0.69 **	−0.35 **	−0.41 **	−0.47 **	−0.13 *	−0.46 **	−0.49 **
Global QoL/health status	−0.49 **	−0.49 **	−0.43 **	−0.27 **	−0.43 **	−0.18 **	−0.42 **	−0.50 **	−0.21 **	−0.18	−0.30 **	−0.17 **	−0.29 **	−0.40 **
Fatigue	0.56 **	0.59 **	0.56 **	0.37 **	0.51 **	0.16 *	0.47 **	0.60 **	0.33 **	0.42 **	0.41 **	0.12	0.40 **	0.47 **
Nausea/vomiting	0.32 **	0.21 **	0.17 *	0.15 *	0.17 *	0.10	0.17 **	0.20 **	0.09	0.26 **	0.08	−0.08	0.18 **	0.24 **
Pain	0.48 **	0.58 **	0.73 **	0.28 **	0.46 **	0.07	0.41 **	0.56 **	0.26 **	0.39 **	0.41 **	0.01	0.43 **	0.41 **
Dyspnoea	0.29	0.38	0.23 **	0.10	0.18 *	0.16 *	0.15 *	0.23 **	0.10	0.13	0.14 *	0.16 *	0.15 *	0.15 *
Sleep/insomnia	0.47 **	0.56 **	0.60 **	0.25 **	0.37 **	0.16 **	0.40 **	0.49 **	0.27 **	0.29 **	0.35 **	0.08	0.34 **	0.34
Appetite loss	0.43 **	0.46 **	0.36 **	0.21 **	0.32 **	0.15 *	0.30 **	0.44 **	0.16 *	0.41 **	0.27 **	−0.04	0.24 **	0.25 **
Constipation	0.15 *	0.26 **	0.20 **	0.05	0.26 **	0.08	0.26 **	0.23 **	0.16 *	0.16	0.15 *	0.05	0.18	0.22 **
Diarrhoea	0.29 **	0.21 **	0.20 **	0.20 **	0.12	−0.003	0.21 **	0.25 **	0.09	0.13	0.19 **	−0.01	0.10	0.16 *
Financial difficulties	0.59 **	0.57 **	0.40 **	0.38 **	0.68 **	0.15 *	0.49 **	0.63 **	0.28 **	0.45 **	0.42 **	0.14 *	0.45 **	0.47 **

<0.40, weak correlation (white), 0.40–0.60, moderate correlation (light grey), and >0.60, high correlation (dark grey); * *p* < 0.05; ** *p* < 0.01. W1: emotional and psychological consequences; W2: physical consequences; W3: pain and discomfort. 1: concerns about condition, 2: job and education; 3: doctor-patient relationship, communication and information; 4: effect of desmoid-type fi-bromatosis (DTF) on relationships; 5: physical limitations and consequences; 6: diagnostic and treatment trajectory of DTF; 7: parents and fertil-ity; 8: body image and sensations; 9: supportive care; 10: concerns around treatment and its consequences: 11: unpredictable course and nature of DTF. Abbreviations: DTF, desmoid-type fibromatosis

## Data Availability

The datasets used and/or analysed during the current study are available from the corresponding author on reasonable request.

## Supplementary Materials

The following are available online at https://www.mdpi.com/article/10.3390/cancers14030709/s1, Table S1: Summary of results of multi-trait scaling new scales. Supplementary Questionnaire: Final 96 items of the DTF-QoL Questionnaire.
