# Supplementary material for: Development of a Disease-Specific Health-Related Quality of Life Questionnaire (DTF-QoL) for Patients with Desmoid-Type Fibromatosis
Source: Cancers (Basel). 2022 Jan 29;14(3):709. doi: 10.3390/cancers14030709 (PMC8833407; doi:10.3390/cancers14030709)
Supplement: Supplementary file 1 [file cancers-14-00709-s001.zip › cancers-1519821-supplementary/cancers-1519821-Supplementary questionnaire.pdf]

cancers MDPI

Development of a Disease-Specific Health-Related Quality of Life Questionnaire (DTF-QoL) for Patients with Desmoid-Type Fibromatosis

Anne-Rose W. Schut, Emma Lidington, Milea J. M. Timbergen, Eugenie Younger, Winette T. A. van der Graaf, Winan J. van Houdt, Johannes J. Bonenkamp, Robin L. Jones, Dirk. J. Grünhagen, Stefan Sleijfer, Cornelis Verhoef, Spyridon Gennatas and Olga Husson

Supplementary Questionnaire. Final 96 items of the DTF-QoL Questionnaire. The questionnaire may be used for free for scientific purposes after permission of the main author. Additionally, the scoring manual can be requested by the main author.

Question	Description	Not at all	A little	Quite a bit	Very much	
	**During the past week:**					
1.	Have you felt uncertain?	1	2	3	4	
2.	Has pain interfered with your sleep at night?	1	2	3	4	
3.	Have you had a bad temper because of the condition?	1	2	3	4	
4.	Have you been unsatisfied with your body?	1	2	3	4	
5.	Have you had problems getting dressed?	1	2	3	4	
6.	Have you felt isolated?	1	2	3	4	
7.	Have you felt disabled?	1	2	3	4	
8.	Have you had pain while sitting?	1	2	3	4	
9.	Have you had stiffness in your limbs?	1	2	3	4	
10.	Have you had any trouble walking?	1	2	3	4	
11.	Have you had swelling in your legs or ankles (oedema)?	1	2	3	4	
12.	Have you been unable to lean on the tumour sites? (e.g., due to local pressure)?	1	2	3	4	
13.	Have you felt lonely?	1	2	3	4	
14.	Have you worried about the disease being aggressive?	1	2	3	4	
15.	Have you worried about dying?	1	2	3	4	
16.	Have you had problems with your appearance?	1	2	3	4	
	**In the last four weeks:**					
17.	Have you had a decreased libido?	1	2	3	4	Not applicable
	**Since your diagnosis:**					
18.	Have you been afraid of tumour growth?	1	2	3	4	
19.	Have you felt there is something in your body that does not belong there?	1	2	3	4	
20.	Has desmoid fibromatosis or its treatment caused those close to you to feel distressed?	1	2	3	4	
21.	Have you been afraid of getting another tumour?	1	2	3	4	
22.	Have you been worried or concerned about the future?	1	2	3	4	
23.	Have you felt a need to keep your fears, concerns and/or symptoms from family members or friends?	1	2	3	4	
24.	Has your physical fitness level reduced?	1	2	3	4	
25.	Have you felt asymmetrical and/or misshapen due to the desmoid fibromatosis or the treatment?	1	2	3	4	Not applicable
26.	Have you felt worried constantly?	1	2	3	4	
27.	Have you been disappointed by the course of your condition?	1	2	3	4	
28.	Have you felt you had to fight this condition?	1	2	3	4	
29.	Have you had problems with confidence?	1	2	3	4	
30.	Have you worried that you are a burden to other people?	1	2	3	4	

Question	Description	Not at all	A little	Quite a bit	Very much	
31.	Have you had difficulties explaining your condition to others?	1	2	3	4	
32.	Do you think your condition is not well understood by people close to you?	1	2	3	4	
33.	Have you felt less independent and/or more dependent on others?	1	2	3	4	
34.	Have you had problems eating?	1	2	3	4	
35.	Has the desmoid fibromatosis had a negative impact on your family life?	1	2	3	4	
36.	Has your physical condition or medical treatment interfered with your social activities?	1	2	3	4	
37.	Have you had extra expenses due to your physical condition or medical treatment (e.g. for medication, transport and/or aids)?	1	2	3	4	
38.	Has your physical condition or medical treatment interfered with your marriage or intimate relationships?	1	2	3	4	Not applicable
39.	Have you had problems with your ability to have children because of your desmoid fibromatosis?	1	2	3	4	Not applicable
40.	Have you felt that you have received less attention from family and friends because the condition is benign?	1	2	3	4	
41.	Have you had problems with your job or your education?	1	2	3	4	Not applicable
42.	Have you felt supported by your family members and/or friends?	1	2	3	4	
43.	Have you had problems driving a car?	1	2	3	4	
44.	Have you lost friendships?	1	2	3	4	
45.	Have you been afraid of needing a limb amputation?	1	2	3	4	Not applicable
46.	Have you been afraid of your desmoid fibromatosis coming back?	1	2	3	4	Not applicable
47.	Have you felt a change in sensation in the area around the tumour?	1	2	3	4	
48.	Have you wanted to cover-up the tumour area and /or scar(s)?	1	2	3	4	
49.	Have you felt addicted to pain medication?	1	2	3	4	Not applicable
50.	Have you lost your hair?	1	2	3	4	
51.	Has your parental role been affected because of your desmoid fibromatosis?	1	2	3	4	Not applicable
52.	Have you felt that doctors are unfamiliar with desmoid-type fibromatosis?	1	2	3	4	
53.	Have you felt stressed around check-ups?	1	2	3	4	
54.	Have you felt frustrated about the "benign" diagnosis with cancerous features?	1	2	3	4	
55.	Have you felt stressed about the diagnosis?	1	2	3	4	
56.	Did you think it took a long time to get a definite diagnosis?	1	2	3	4	
57.	Have you felt like a cancer patient?	1	2	3	4	
58.	Have you felt reassured by the benign nature of your disease?	1	2	3	4	
59.	Do you think your prognosis (the expected improvement or worsening of your condition) is clear?	1	2	3	4	
60.	Have you felt there is no optimal treatment for you?	1	2	3	4	
61.	Have you felt your desmoid fibromatosis has changed your life in a negative way?	1	2	3	4	
62.	Have you wanted to meet others with desmoid fibromatosis?	1	2	3	4	
63.	Have you had doubts about the effectiveness of your treatments?	1	2	3	4	Not applicable
64.	Have you found it frustrating having to explain your condition to others?	1	2	3	4	
65.	Have you lacked information about your desmoid fibromatosis and/or its treatment?	1	2	3	4	
66.	Have you been satisfied with your communication with your professional(s)?	1	2	3	4	

Question	Description	Not at all	A little	Quite a bit	Very much	
67.	Have you had to explain your circumstances to others?	1	2	3	4	
68.	Do you feel like you have a chronic disease?	1	2	3	4	
69.	Have you lacked psychological support?	1	2	3	4	
70.	Have you had to take sick leave?	1	2	3	4	Not applicable
71.	Have your career ambitions changed because of the diagnosis?	1	2	3	4	Not applicable
72.	Have you experienced a lack of continuity (seeing the same doctors / specialised nurse) in the care for your desmoid fibromatosis?	1	2	3	4	
73.	Have you had problems receiving enough information about your desmoid fibromatosis and its treatment?	1	2	3	4	
74.	Have you been bothered by long travel to the hospital?	1	2	3	4	
75.	Have you worried about passing the condition to your children?	1	2	3	4	Not applicable
76.	Have you had a problem receiving treatments in a cancer hospital?	1	2	3	4	
77.	Have you worried about not being able to continue working or studying?	1	2	3	4	Not applicable
78.	Has the colour of your hair changed?	1	2	3	4	Not applicable
79.	Have you felt that you are standing still in life?	1	2	3	4	
80.	Have you felt less feminine/masculine?	1	2	3	4	
81.	Have you received different contradictory recommendations about treatment options?	1	2	3	4	
82.	Have you had to change jobs as a result of your condition?	1	2	3	4	Not applicable
83.	Have you felt you were wasting the time of cancer specialists?	1	2	3	4	
84.	Have you worried about your ability to have children?	1	2	3	4	Not applicable
85.	Were you frightened by the referral to the cancer hospital?	1	2	3	4	
86.	Have you lacked online support (forum and/or chat group)?	1	2	3	4	
87.	Has there been mutual trust between you and your professional(s)?	1	2	3	4	
88.	Have you felt embarrassed using mobility aids (e.g. wheelchair, scooter, electric bike)?	1	2	3	4	Not applicable
89.	Have you had a rash as a result of the treatment?	1	2	3	4	
90.	Have you worried about your treatment?	1	2	3	4	
91.	Have you worried about a decrease in muscle strength after treatment?	1	2	3	4	
92.	Have you received enough information about the possible treatment side effects?	1	2	3	4	
93.	Have you been worried about tumour growth during pregnancy?	1	2	3	4	Not applicable
94.	Were you passed from one hospital to another before the final desmoid fibromatosis diagnosis?	1	2	3	4	
95.	Have you worried about being treated unfairly because of your desmoid fibromatosis (i.e. at work, by insurance companies)?	1	2	3	4	
96.	Have you worried about being able to access treatments in the future?	1	2	3	4	Not applicable

Scales of the DTF-QoL

	Scale	Question Number
W1	Emotional and psychological consequences	1, 3, 4, 6, 13, 14, 15, 16
W2	Psychological consequences	5, 7, 9, 10, 11
W3	Pain and discomfort	2, 8, 12
1	Concerns about condition	18, 20, 21, 22, 26, 53, 55, 90
2	Job and education	41, 70, 71, 77, 82
3	Doctor-patient relationship, communication and information	65, 66, 72, 73, 87, 92
4	Effect of DTF on relationships	23, 29, 30, 31, 32, 35, 38, 40, 42, 64, 67, 79, 95
5	Physical limitations and consequences	24, 33, 34, 36, 37, 43, 44, 88
6	Diagnostic and treatment trajectory of DTF	52, 56, 74, 76, 81, 85, 94
7	Parents and fertility	39, 51, 75, 84, 93
8	Body image and sensation	25, 47, 48, 80
9	Supportive care	62, 69, 86
10	Concerns around treatment and its consequences	45, 49, 63, 91
11	Unpredictable course and nature of DTF	19, 27, 28, 46, 54, 57, 58, 59, 60, 68, 96
	**Single items**	
	Decreased libido	17
	Hair loss	50
	DTF changed life in negative way	61
	Hair colour	78
	Wasting time of cancer specialists	83
	Rash treatment	89

Abbreviations: DTF, desmoid-type fibromatosis.
