# Supplementary material for: Development of a Disease-Specific Health-Related Quality of Life Questionnaire (DTF-QoL) for Patients with Desmoid-Type Fibromatosis
Source: Cancers (Basel). 2022 Jan 29;14(3):709. doi: 10.3390/cancers14030709 (PMC8833407; doi:10.3390/cancers14030709)
Supplement: Supplementary file 1 [file cancers-14-00709-s001.zip › cancers-1519821-supplementary/cancers-1519821-Supplementary table.pdf]

cancers

MDPI

Development of a Disease-Specific Health-Related Quality of Life Questionnaire (DTF-QoL) for Patients with Desmoid-Type Fibromatosis

Anne-Rose W. Schut, Emma Lidington, Milea J. M. Timbergen, Eugenie Younger, Winette T. A. van der Graaf, Winan J. van Houdt, Johannes J. Bonenkamp, Robin L. Jones, Dirk. J. Grünhagen, Stefan Sleijfer, Cornelis Verhoef, Spyridon Gennatas and Olga Husson

Table S1. Summary of results of multi-trait scaling new scales.

					Item-scale convergent validity Criterion 1 (inclusive criterion)		Item-scale divergent validity Criterion 2 (exclusive criterion)		Scaling fulfilment
	New scales	*n*	Cronbach's alpha old scale	Cronbach's alpha new scale	Range of item-scale correlations [4]	Number of item-scale correlations [5]	Range of correlations with other scales [6]	Number of items higher correlation with other scale [7]	Number of items that meet criterion 1 but not 2
	**Scale 5 and 10 [1]**								
5	Physical limitations and consequences (Item 36; 40; 39; 27; 48; 49; 37)	7	0.868 *	0.836 *	0.323–0.742	6/7	0.046–0.731	2/7	4/7
10	Concerns around treatment and its consequences (Item 50; 55; 69; 94; 97)	5	0.551	0.697	0.334–0.576	3/5	0.003–0.603	4/5	1/5
	**Scale 6 and 1 [2]**								
6	Diagnostic and treatment trajectory of DTF (Item 80; 82; 100; 62; 58; 87)	6	0.680 *	0.683 *	0.262–0.553	3/6	0.090–0.489	2/6	3/6

1	Concerns about condition (Item20; 24; 25; 29; 61; 96; 23; 59; 91)	9	0.893	0.880 *	0.350–0.769	8/9	0.028–0.719	0/9	8/9
	**Scale 8 and 10** [3]								
8	Body image and sensation (Item 53; 86; 28)	3	0.755 *	0.756	0.527–0.665	3/3	0.001–0.711	1/3	2/3
10	Concerns around treatment and its consequences (Item 50; 52; 55; 69; 97)	5	0.551	0.644	0.318–0.465	3/5	0.003–0.621	4/5	1/5

* Cronbach's alpha will improve if one of the items would be deleted

1. Removing item 94 ('Have you worried about your treatment?') improved Cronbach's alpha of scale 5. Item 94 was moved from scale 5 to scale 10. Cronbach's alpha of the new scale 5 did not improve; Cronbach's alpha of new scale 10 showed little improvement, however scaling fulfilment did not improved. Based on these results and content validity we have decided to keep item 94 in scale 5.
2. Removing item 91 ('Were you frightened by the referral to the cancer hospital?') improved Cronbach's alpha of scale 6. Item 91 was moved from scale 6 to scale 1. Based on content validity and impairment of Cronbach's alpha of both new scales, we have decided to keep item 91 in scale 6.
3. Removing item 52 ('Have you felt a change in sensation in the area around the tumour?') improved Cronbach's alpha of scale 8. Item 52 was moved from scale 8 to scale 10. Both Cronbach's alpha coefficients, as well as the scaling fulfilment of the new scales improved. Based on these results and good content validity, we recommend to move item 52 from scale 8 to scale 10.
4. Pearson correlation between items and hypothesized scale (corrected for overlap).
5. Number of item-scale correlations that meet minimum standard for convergent validity.
6. Pearson correlations between items and other scales.
7. Correlations higher between items and other scales in comparison with hypothesized scale.
